# Biological Compatibility of a Polylactic Acid Composite Reinforced with Natural Chitosan Obtained from Shrimp Waste

**DOI:** 10.3390/ma11081465

**Published:** 2018-08-18

**Authors:** Yaret Gabriela Torres-Hernández, Gloria Michel Ortega-Díaz, Lucía Téllez-Jurado, Nayeli Shantal Castrejón-Jiménez, Alejandro Altamirano-Torres, Blanca Estela García-Pérez, Heberto Balmori-Ramírez

**Affiliations:** 1 Department of Metallurgical and Materials Engineering, Escuela Superior de Ingeniería Química e Industrias Extractivas (ESIQIE), Instituto Politécnico Nacional, Unidad Profesional Adolfo López Mateos (UPALM), Av. Instituto Politécnico Nacional S/N, C.P., Ciudad de México 07738, Mexico; ltellezj@ipn.mx (L.T.-J.); hbalmori@ipn.mx (H.B.-R.); 2 Escuela Nacional de Ciencias Biológicas (ENCB), Department of Microbiology, Instituto Politécnico Nacional, Prolongación de Carpio y Plan de Ayala S/N, Casco de Santo Tomás. C.P., Ciudad de México 11340, Mexico; belimax_pelusa@hotmail.com (G.M.O.-D.); naye_nice85@hotmail.com (N.S.C.-J.); abrilestela@hotmail.com (B.E.G.-P.); 3 Department of Materials, Universidad Autónoma Metropolitana-Azcapotzalco, San Pablo No.180, Col. Reynosa-Tamaulipas, C.P., Ciudad de México 02200, Mexico; aat@correo.azc.uam.mx

**Keywords:** biocomposites, polylactic acid, chitosan, osteoblasts, cellular viability, biomineralization

## Abstract

The aim of this work is to evaluate the effect of chitosan content (1, 3 and 5 wt %) dispersed in polylactic acid (PLA) on the structure and properties of composites. Also, the hydrolytic degradation, and the cell viability and adhesion of human MG-63 osteoblasts are analyzed to determine the composites’ suitability for use in tissue engineering. For the manufacture of the materials, natural chitosan was extracted chemically from shrimp exoskeleton. The composites were fabricated by extrusion, because it is a low-cost process, it is reproducible, and it does not compromise the biocompatibility of the materials. FT-IR and XRD show that the chitosan does not change the polymer structure, and interactions between the composite components are discarded. In vitro degradation tests show that the composites do not induce significant pH changes in phosphate buffer solution due to their low susceptibility to hydrolytic degradation. The adhesion and morphological characteristics of the osteoblasts are evaluated using confocal microscopy and scanning electron microscopy. The cell viability is determined by the MTT assay. Osteoblasts adhesion is observed on the surface of PLA and composites. A higher amount of chitosan, higher number of cells with osteoblastic morphology, and mineralized nodules are observed on the composite surface. The highest metabolic activity is evidenced at 21 days. The results suggest that the Polylactic acid/chitosan composites are potentially suitable for use as a biomaterial.

## 1. Introduction

The development of materials that combine the mechanical properties, chemical stability, and biological characteristics required to promote tissue growth is the main focus of research in the field of tissue engineering. A biomaterial must be biocompatible, biodegradable, and able to stimulate cell adhesion, cell proliferation, and in the case of stem cells, differentiation and maturation. The biomaterials must allow the normal formation of new bone tissue, not having as a final goal an implant material but a new, native bone [1], and its degradation products must produce minimal immune reactions. On the other hand, the mechanical properties are one of the most difficult requirements to satisfy, since the scaffold must withstand the mechanical demands of the injured site throughout the regeneration process, implying that the mechanical properties and the degradation mechanisms must work in equilibrium with the formation of new tissue [2,3].

Common biodegradable polymers used in tissue regeneration are polyglycolic acid (PGA), polylactic acid (PLA), PGA-PLA copolymers, polyhydroxyalkanoates (PHA) and polycaprolactone (PCL) [4,5,6]. The linear aliphatic thermoplastic polyester PLA is derived from renewable resources, with wide availability and easy processability by injection molding or extruding [6,7,8,9]. PLA has a high modulus of elasticity (3.5–3.8 GPa) and tensile strength (48–110 MPa); however, the inherent brittleness and low toughness limit its applications [7,8,9]. Because of its biodegradability, biocompatibility, and osteoconductive properties, it has been successfully used in drug delivery systems and in fracture fixation devices, but its hydrophobic surface produces an inflammatory response, and its degradation causes a decline of cell adhesion and cell proliferation [10]. The polymer blends, such as PLA-PLC, PLA-phosphates, PLA-PLGA, PLA-hydroxyapatite, and PLA-chitosan [11,12,13,14,15], are focused on obtaining useful properties. The chitosan blends have a great potential in tissue engineering because chitosan is a biocompatible polymer with a similar structure to the glucosamine of the extracellular matrix. It shows antimicrobial activity, and its hydrophilic surface promotes adhesion, proliferation, and cell differentiation. It has been successfully used for wound healing, promotion of bone growth, and to enhance the anti-inflammatory response [16,17].

Natural chitosan is obtained by deacetylation of chitin, which is the main component of the exoskeleton of crustaceans, arthropods, and cell walls of some fungi [18,19,20,21]. However, due to its low mechanical properties [1,22], it is necessary to combine it with other synthetic or natural polymers. Some investigations about PLA-chitosan composites are described below. Chitosan microspheres with Bone Morphogenic Proteins (BMPs) have been dispersed into a PLA matrix in order to obtain PLA-chitosan composites by thermally induced phase separation [23]. The results from this research show that the introduction of different contents of microspheres improves the composites’ compression strength. In vitro degradation tests reveal that the degradation of chitosan is faster than that of PLA; therefore, the authors suggest that the composite offers a novel scaffold design for non-loaded bone regeneration. In another research, polylactide/chitosan blend membranes were obtained by solvent casting, and it was found that the two components interact, probably due to hydrogen bonds [24]. However, the use of organic solvents in the two processes mentioned could compromise the composites biocompatibility. The fabrication by vacuum reaction of a scaffold through grafting of lactic acid on the amino groups in chitosan, and the in vitro (lysozyme/PBS solution) and in vivo (Kunming mice) degradation showed that the mass loss is faster in vivo than in vitro, and that PLA/chitosan copolymers have better histocompatibility than chitosan or PLA alone [22]. Physicochemical and antimicrobial properties have also been studied in a PLA film with 5 and 10% of chitosan and two particle sizes (715 and 180 µm) fabricated by extrusion [25]. The main results from this research show that the incorporation of chitosan particles decreases the stiffness and extensibility of the films. The thermal behavior of the PLA is not affected by chitosan due to the low ratio of chitosan in the composites, and the water vapor permeability is higher than for pure PLA films. The antimicrobial activity decreases in the presence of chitosan. Finally, methods such as 3D printing [26], gel casting, and stereolithography require costly equipment, the manufacture time is long, and the material is required in powder. In gel casting and stereolithography, it is necessary to employ polymers which are compatible with UV rays [26,27,28].

As previously mentioned, the advantage of composite materials is to improve or combine the properties of the individual components in order to obtain a new material with the best properties according to the requirements. Chitosan has a similar structure to the glucosamine of the extracellular matrix, and improves the cells adhesion, proliferation, and differentiation; however, due to its low mechanical properties, it is necessary to combine it in a PLA matrix that has better mechanical properties, and then to reduce the inflammatory responses caused by acid products of the hydrolytic degradation process [10,14]. The composites formed with PLA matrix that incorporates chitosan particles present an alternative biomaterial, due to their properties when they are combined.

In this work the aim is to combine the best properties of both PLA and chitosan in order to obtain a biomaterial with an adequate degradation rate, as well as to regulate bone cell functions, such as adhesion and proliferation. The employed chitosan was extracted from shrimp waste, and PLA/chitosan composite bars have been fabricated without the use of solvents by extrusion, which is a process that does not compromise the biocompatibility; these factors reduce the cost of the production of the material.

## 2. Experimental Procedures

### 2.1. Materials and Methods

The polylactic acid (PLA, 2002D, Nature Works^®^, Minnetonka, MN, USA) used had 4.25% of isomer D content, molecular weight of 212 KDa, polydispersity index of 3.06, specific gravity of 1.24 (ASTM D256), and melt index 5–7 (ASTM D1238), [29]. The chitosan used as filler was extracted from white shrimp exoskeletons (*Litopenaus Vannamei*), and it had an 80% of deacetylation degree and average particle size of 300 µm.

#### 2.1.1. Extraction of Chitosan

The chitosan was extracted from shrimp waste by a chemical treatment method [18,19,20,21], described as follows. Four kilograms of shrimp exoskeletons were washed with water, and oven-dried at 80 °C for 4 h. The washed wastes were ground in an agate mortar, and then subjected to a chemical treatment in a 1% NaOH solution at 28 °C for 24 h, with magnetic stirring for removal of proteins. After that, the minerals were removed in a 0.6 N HCl solution, with a 1:11 solid-liquid ratio at 30 °C for 3 h. The deacetylation process was carried out by adding 50% of NaOH solution with a 1:11 solid–liquid ratio at 60 °C for 2 h. A discoloration process was made with a solution of 15% ether, 75% acetone, and 10% distilled water at 65 °C for 2 h, with magnetic stirring. Finally, the sample was exhaustively washed with distilled water. All chemical reagents used were reagent grade.

#### 2.1.2. Composites Preparation

The chitosan particles were mixed with PLA pellets in an α-alumina cylindrical container without milling balls; the mixtures were processed for 2 h at 200 rpm. The composite’s nomenclature was PLA, PLA/Q1, PLA/Q3, and PLA/Q5 for composites with 0, 1, 3 and 5 wt % of chitosan particles, respectively. The composites were fabricated by extrusion in a single-screw extruder (Beutel Spacher, Mexico City, Mexico) with 24:1 Length:Diameter ratio, at 150 °C with a rotor speed of 20 rpm, with a profile extrusion die that creates manufactured specimens for mechanical testing. Previous to the extrusion, the mixtures of PLA and chitosan were dried in a furnace at 80 °C for 6 h.

### 2.2. Microstructural and Mechanical Characterization 

The PLA and chitosan particle morphology, as well as the chitosan dispersion in the polymeric matrix, were characterized by Confocal Laser Microscopy (Confocal Multiphotonic Microscope, Carl Zeiss, LSM 710 NLO, Jena, Germany), using an excitation wavelength of 400 nm with an emission range of 400–800 nm. The specimen’s crystallinity was analyzed by X-ray diffraction (XRD) in a Focus, D-8, Bruker (Billerica, MA, USA) diffractometer, using Cu-kα radiation. The diffraction patterns were recorded from 5° to 40° in 2θ, with a step wise of 0.05°. The functional group identification, and interactions between the composite components were studied by Fourier Transform Infrared Spectroscopy (FT-IR, Perkin Elmer, Waltham, MA, USA, model Spectrum One). The spectra were recorded in the middle infrared range of 4000–400 cm^−1^, in transmission mode at intervals of 2 cm^−1^ and 16× scanning. The biomaterial’s thermal behavior was studied using a SDTQ 600, TA Instrument (New Castle, DE, USA) in the temperature range of 25–500 °C, heating at 5 °C/min in controlled nitrogen. For the mechanical tests, the specimens were cut longitudinally to the extrusion direction, the tensile tests (ASTM D-638 [30]) were performed at room temperature using a Universal Testing Machine (United, model: SSTM-1) with a load cell of 10 kN and cross–head speed of 0.2 in/min. Ten specimens of each composite composition were used.

### 2.3. Biocompatibility Analysis

#### 2.3.1. In Vitro Degradation in PBS

The materials’ in vitro degradation was measured using a phosphate buffer solution (PBS, 3813, Sigma-Aldrich, St. Louis, MO, USA). Samples of 9 mm × 10 mm × 4 mm were weighed before placing them in 10 mL vials with PBS, at a pH = 7.4 ± 0.1 at 37 °C by predetermined periods of time (1–28 days). The change in pH was measured using a pH meter (Radiometer Analytical SAS, Lyon, France, model pH 220). The reported pH value is the average of three samples.

#### 2.3.2. Cell Culture

The human osteoblast cell line MG-63 (ATTC CRL-1427) was cultured in α-Minimal Essential Medium (MEM, Thermo Scientific, Waltham, MA, USA) supplemented with 10% heat-inactivated fetal bovine serum (FBS, Gibco, Thermo Fisher Scientific) and antibiotics (gentamicin-penicillin, 25 mg/L and 50,000 U/L, respectively), at 37 °C in a 5% CO_2_ atmosphere. Media was renewed every three days, and when the cells reached confluence, a trypsin-EDTA solution (0.05 g/L trypsin and 0.05 g/L EDTA) was used to detach the cells from culture flasks.

##### Cell Culture on Biomaterials

The PLA and composites were sterilized by immersion in a 70% ethylic alcohol solution and by exposition to UV-light for 30 min. For all experiments, sterilized biomaterials were incubated with α-MEM overnight, and were then transferred to a 96-well sterile culture plate. MG-63 cells were cultured at 5 × 10^4^ cells/biomaterial of density, and maintained in a humidified atmosphere of 5% CO_2_ at 37 °C. Culture media was changed daily and the cytoskeletal arrangement, cell adherence, and cell viability was measured on days 1, 7, 14, and 21.

##### Actin Stain

To evaluate the osteoblast morphology on biomaterials, immunofluorescence assays were conducted to analyze actin distribution. MG-63 osteoblast-like cells were seeded onto sterilized biomaterials at a density of 5 × 10^4^ cells/biomaterial for 1, 7, 14 and 21 days, and incubated at 37 °C and 5% CO_2_. After each time, the samples were fixed with 4% paraformaldehyde (Sigma-Aldrich), followed by washing three times with PBS. Actin filaments were stained with rhodamine-phalloidin (10 ng/µL, Sigma-Aldrich) for 20 min at room temperature, and the excess of rhodamine was eliminated by washing five times with PBS. Preparations were viewed in a confocal laser scanning microscope system (LSM5 Pascal, Zeiss).

##### Scanning Electron Microscopy

MG-63 osteoblasts grown on PLA and composites for 1, 7, 14, and 21 days were fixed with 2.5% glutaraldehyde and post-fixed with 1% aqueous osmium tetroxide. Afterwards, cells were washed three times, followed by dehydration with increasing concentrations of ethanol and critical point dried with liquid CO_2_. The preparations were coated with gold (Denton Vacuum-Desk II, Moorestwon, NJ, USA) and observed using a scanning electron microscope (JSM 7800, JEOL, Tokyo, Japan).

##### Cell Viability

To evaluate the cell viability of the osteoblasts that grew in the PLA and composites, MTT (3-[4,5-dimethylthiazol-2-yl]-2,5-diphenyltetrazolium bromide; thiazolyl blue) assay was performed. For that, osteoblasts were cultured at a density of 50,000 cells/biomaterial and grown at 37 °C and 5% CO_2_ for 24 h. After that period, the biomaterials were transferred to a new plate to permit the growth of cells that were attached to the surface. After 1, 7, 14, and 21 days, the media was removed and replaced for 100 µL of 100 mg/mL MTT solution (Sigma). The reaction was allowed to occur for 3 h at 37 °C. After that time, 100 µL of dimethyl sulfoxide were added to dissolve the precipitate. The absorbance was read at 590 nm using an ELISA plate reader (Thermo Fisher Scientific, Multiskan GO).

The osteoblasts viability on the PLA and composites was confirmed by confocal microscopy analysis. For that, 50,000 osteoblasts were cultured on biomaterials and the growing kinetics was followed for 1, 7, and 14 days. After each time, the cells attached on the biomaterials were stained with a mix of nucleic acid dyes, Syto9 (5 µmol/L; Invitrogen, Carlsbad, CA, USA) and propidium iodide (PI; 30 µmol/L; Sigma-Aldrich), and analyzed by confocal microscopy. Three experiments of each incubation time were measured and their standard deviation was calculated. Statistically significant differences were determined by comparing the optic density of 7, 14, and 21 days with the optic density of 1 day. The variance (ANOVA) was calculated with the Sigma Stat software (3.5), *P* values < 0.05 were considered statistically significant.

##### Alizarin Red Stain

The cells that grew on the PLA and composites were fixed with 4% paraformaldehyde for 15 min at room temperature. Then, the cells were washed 3 times with PBS, and 200 µL of 2% Alizarin red were added to each sample. Cells were incubated during 20 min with gentle agitation, and washed 5 times with PBS. Finally, the cells were analyzed under an inverted microscope (Primovert, Zeiss).

## 3. Results and Discussion

### 3.1. Distribution of Chitosan in PLA

**Figure 1:**
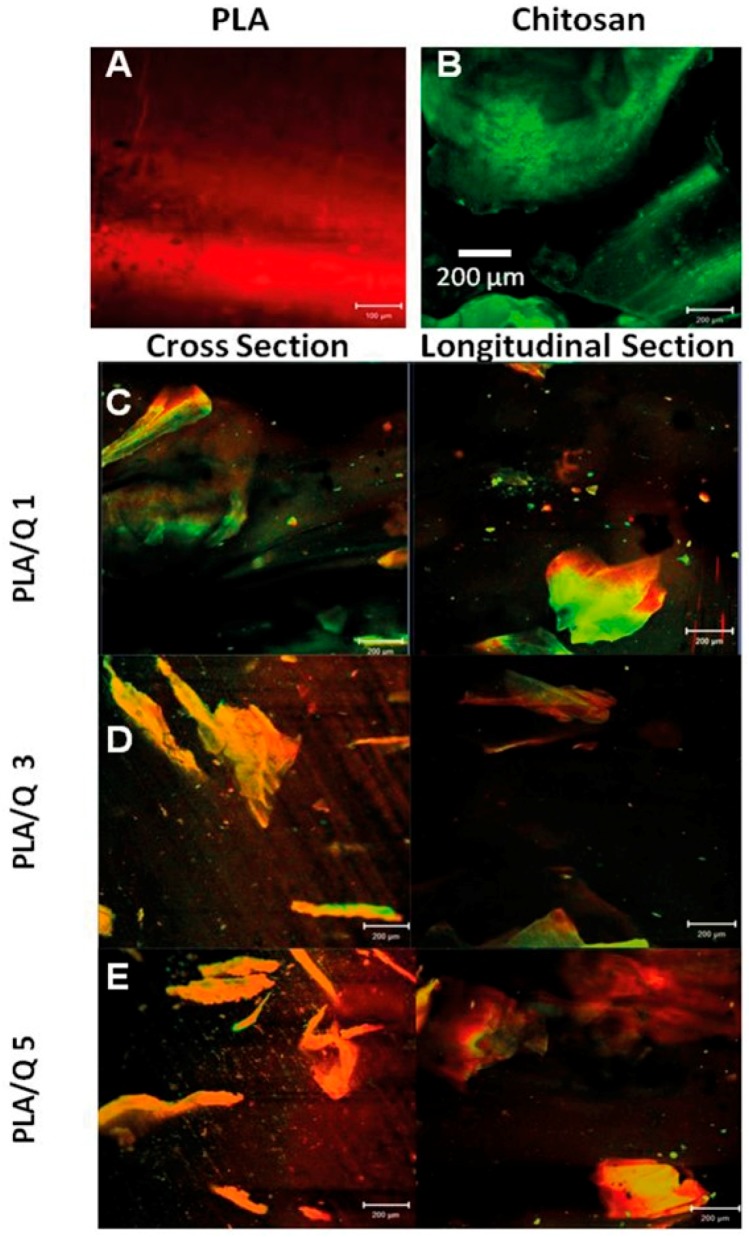
Confocal microscopy micrographs of (**A**) Polylactic acid (PLA), (**B**) Chitosan particles, and (**C**–**E**) PLA/chitosan composites. The PLA and chitosan particles show red (**A**) and green (**B**) autofluorescence, respectively.

The chitosan particle distribution in the composites matrix was evaluated by confocal microscopy (Figure 1). The images show that the chitosan particles for the composites with 1 and 3 wt % chitosan were evenly distributed in the , and that they have flake morphology with lengths of up to 500 µm. The composite with 5 wt % chitosan showed some agglomerates. The average particle size and the morphology of the particles, as well as the processing method of the composites, have a great influence in the homogeneity and dispersion of the particles in the matrix [8,29]. The PLA/Q1 and PLA/Q3 composites show a good dispersion of chitosan in the PLA matrix, although the particles are aligned along the extrusion direction because of their flake morphology. The PLA/Q5 composite shows the presence of agglomerates that result in poor adhesion with the PLA matrix.

### 3.2. Structural Characterization of the PLA/Chitosan Composites

**Figure 2:**
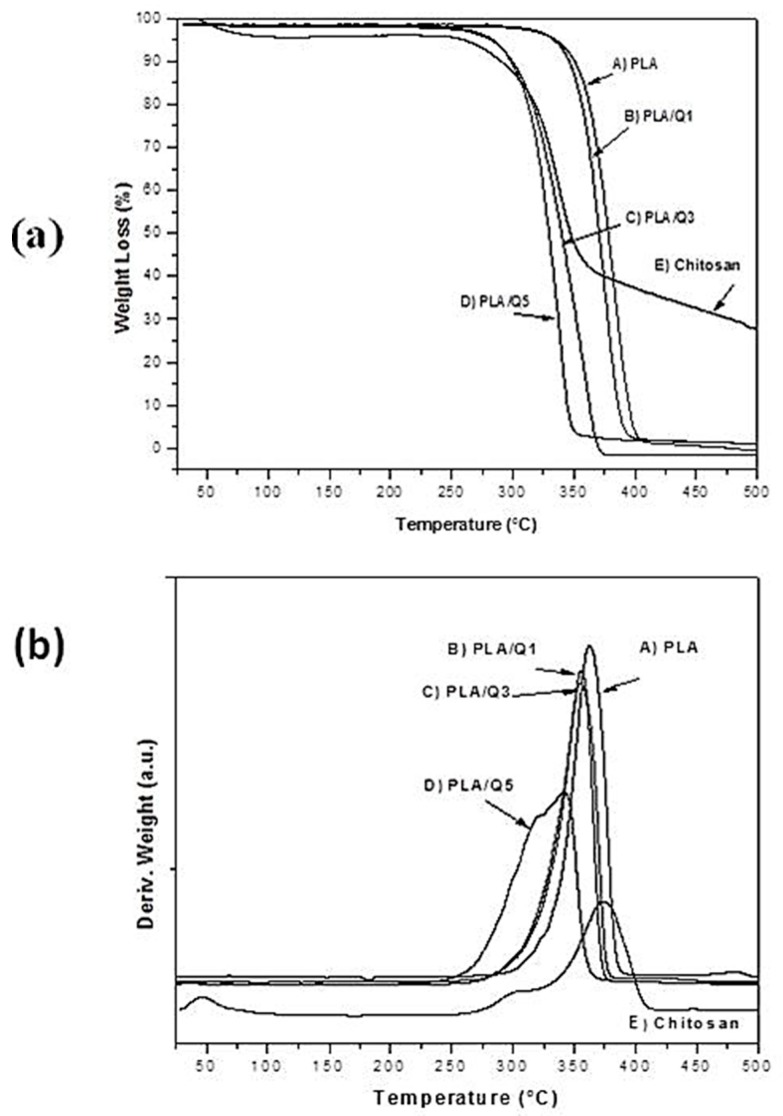
(**a**) Thermogravimetric (TG) and (**b**) TG derivative curves for PLA, chitosan and PLA/chitosan composites with 1 wt % (PLA/Q1), 3 wt % (PLA/Q3) and 5 wt % (PLA/Q5) chitosan.

The thermal behaviors of PLA, chitosan, and composites were evaluated by thermogravimetric (TG) analysis, and the weight losses during heating and their derivatives are shown in Figure 2a,b, respectively. The TG curve of chitosan exhibits three decomposition stages. The first occurs from room temperature to ~87 °C with ~5 wt % loss; it can be attributed to the evaporation of adsorbed water. The second stage starts at 250 °C, with a maximum weight loss rate at ~300 °C, but it is overlapped with the beginning of the third stage, that presents a maximum weight loss rate at 390 °C that continues above 500 °C. The total weight loss of both stages is ~75%; this is due to the degradation process which includes the dehydration of saccharide rings and the polymerization and decomposition of acetylated and deacetylated units of chitin. For PLA, there is one weight loss stage between 300 and 390 °C, with a maximum rate at 357 °C, and a total weight loss of ~95%, that is related to the breaking of ester bonds and the release of gaseous products such as cyclic oligomers, lactide molecules, acetaldehyde, and carbon monoxide [31]. The PLA/chitosan composites present two stages of decomposition. In comparison with the PLA matrix thermal decomposition, the composite’s decomposition temperatures decrease with increasing the amount of chitosan particles. In the PLA/Q5 composite, the first decomposition step starts at ~230 °C, and has a maximum weight loss rate at ~320 °C. These two temperatures roughly correspond with chitosan’s first decomposition stage, so this is probably related to the decomposition of chitosan in the composite. The PLA/Q5 composite shows a second decomposition event that has a maximum rate at ~340 °C; this temperature is lower than the maximum decomposition rate temperature for PLA and chitosan alone; therefore, it is reasonable to assume that the decomposition of the PLA and chitosan composite components occurs simultaneously, and that the liberated decomposition products accelerate the decomposition reaction of both.

The thermal behavior of the PLA and PLA/chitosan composites discard a thermal decomposition of the components during the extrusion at 150 °C that was implemented to make them. The observed thermal events agree with those reported in the literature [6,25]. The two decomposition stages observed in the PLA/chitosan composites are related to the decomposition of chitosan, and the simultaneous decomposition of PLA and chitosan in the composite. Also, the presence of chitosan in the PLA matrix decreases the thermal decomposition temperature of the composites as the chitosan amount increases. The PLA shows a thermal degradation temperature ~300 °C, and the composite with 5 wt % chitosan shows a ~50 °C temperature decrease. This is related to the low degradation temperature of chitosan which is observed at ~250 °C, as is reported by some researches, which occurs in the range of 220–260 °C [32], depending on the source of the chitosan.

The decomposition products of chitosan, such as small polar molecules and H_2_O from opening of the pyranose ring (dehydration of saccharide rings, and the polymerization) and deamination reactions (decomposition of acetylated and deacetylated units of chitin), break down the PLA polyester chain, promoting a decrease in the composite’s degradation temperature. Similar results have been reported by other authors [33] using hydrophilic fillers, such as cellulose, in PLA matrix.

**Figure 3:**
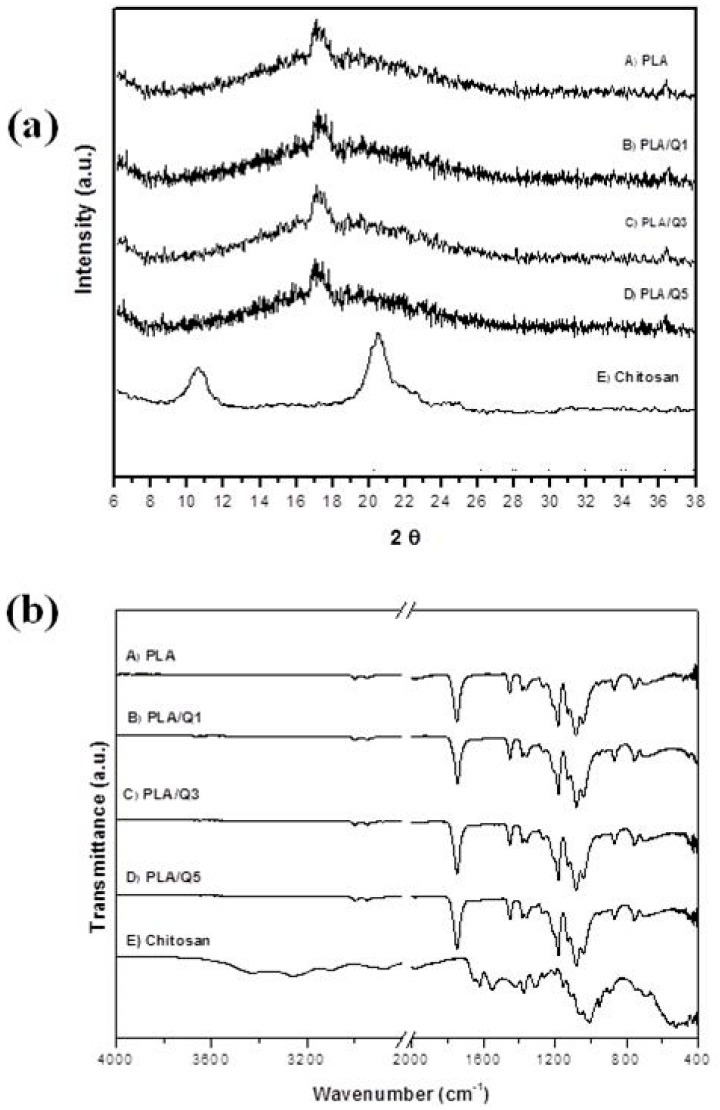
(**a**) X-ray diffraction patterns and (**b**) FT-IR spectra of A) PLA; PLA/chitosan composites with B) 1 wt % (PLA/Q1), C) 3 wt % (PLA/Q3), D) 5 wt % (PLA/Q5) chitosan, and E) chitosan.

The material’s crystallinity was analyzed by XRD (Figure 3a). The XRD pattern of PLA shows a small, broad peak at 17.20° in 2θ. The chitosan pattern shows two peaks at 10.2° and 20.5° in 2θ. The composite’s diffraction patterns were similar to the PLA pattern. The crystallinity of chitosan depends on its extraction source. The chitosan obtained from shrimp exoskeletons by chemical treatment has a high crystallinity degree because the intermediate product is α-chitin, which produces chitosan with higher crystallinity; other sources produce β-chitin, yielding chitosan with less crystallinity [18,19,31]. In our work, poor crystallinity was observed, since only a small and broad peak in the biocomposites XRD patterns, corresponding to the characteristic pattern of PLA [7,8], was observed. The chitosan signal in the patterns was not observed because the amount of chitosan was below the technique detection limit. 

The functional groups of PLA, chitosan, and composites were identified by FT-IR; the spectra are shown in Figure 3b. The FT-IR spectrum of PLA shows several characteristic bands: at 1744 cm^−1^ it shows a strong band due to the C=O stretching vibration of the ester group and the bending vibration of this group appears at 1180 cm^−1^; the band at 866 cm^−1^ corresponds to the amorphous phase, and at 754 cm^−1^ appears the band that corresponds to the crystalline phase of PLA [7,8]. The chitosan spectrum shows bands at 3256 and 3104 cm^−1^ due to O–H and N–H stretching vibrations. The bands at 1659 and 1548 cm^−1^ are attributed to C=O stretching vibrations of the amide group (I and II). The stretching vibrations of C–H bond in C–H_2_ are located at 2868 cm^−1^, the bands at 890 and 1152 cm^−1^ correspond to wagging of the saccharide unit of chitosan, and the bands at 1069–1021 cm^−1^ are attributed to CH_3_ bending vibration [32,34]. The bands at 2996 cm^−1^ and 2943 cm^−1^ are assigned to the C–H stretching vibration. The FT-IR spectra of the composites are similar to the PLA spectrum.

The interaction of chitosan and PLA depends on the components, chemical structure, and the processing method used to integrate them. When the materials are fabricated by grafting lactic acid onto chitosan’s amino group, the carbonyl group of PLA interacts with the hydroxyl groups in the structure of chitosan, creating ester and amide bonds which promote a displacement of the absorption bands in the FT-IR spectra and the presence of a new band at ~1728 cm^−1^ that corresponds to the ester bond [22]. On the other hand, the two components do not interact when chitosan particles are only dispersed in the PLA matrix [25], as can be seen in our work.

### 3.3. Mechanical Properties of the PLA/Chitosan Composites

**Figure 4:**
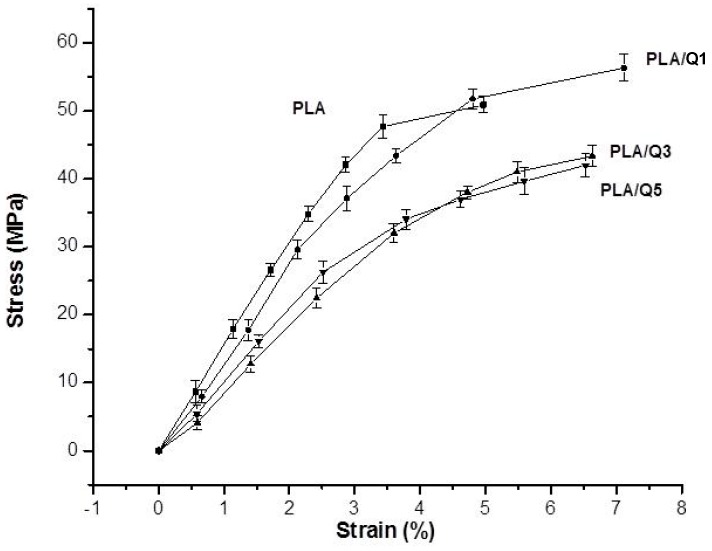
Stress-strain curves in tension of PLA and PLA/chitosan composites with 1 wt % (PLA/Q1), 3 wt % (PLA/Q3) and 5 wt % (PLA/Q5) chitosan.

The mechanical properties of PLA and PLA/chitosan composites were investigated to find out the effect of chitosan in the PLA matrix. Typical stress-strain curves in tension are shown in Figure 4, and the relevant properties are given in Table 1. For PLA, the behavior is brittle, and the addition of chitosan improves the ductility. Regarding the effect of chitosan on the ultimate tensile strength, it increases ~14% for the PLA/Q1 composite, but it decreases for higher chitosan contents. The modulus of elasticity decreases as the percentage of chitosan increases.

The PLA/Q1 composite has a higher tensile strength (56.34 ± 2.64 MPa) than PLA (49.36 ± 4.44 MPa); this is due to a good dispersion of the particles in the matrix. The increase of the amount of particles impairs the homogeneous distribution and the interfacial adhesion, and favors the formation of chitosan agglomerates. This effect reduces the tensile strength of PLA/Q3 (43.38 ± 3.29 MPa) and PLA/Q5 (42.19 ± 2.55 MPa) composites. However, the tensile strengths of the PLA/chitosan composites are within the range of properties of a variety of tissues, and fulfill the requirements for biomaterial applications [35,36,37]. The decrease of the composites modulus of elasticity is due to the lower modulus of chitosan, and for the composites with 3 and 5 wt %, it is also due to the formation of chitosan agglomerates and weak interfacial adhesion at the chitosan-PLA interface.

### 3.4. Biocompatibility of the PLA/Chitosan Composites

The degradability and biocompatibility of the PLA/chitosan composites were studied through a series of experiments that involved following the changes in pH as a function of time when the composites were submerged in PBS medium, as well as the proliferation and adhesion of the human osteoblast cell line MG-63 (ATTC CRL-1427) on the composites surface. The results are presented in the following paragraphs.

#### 3.4.1. Hydrolytic Degradation of Composites in PBS

**Figure 5:**
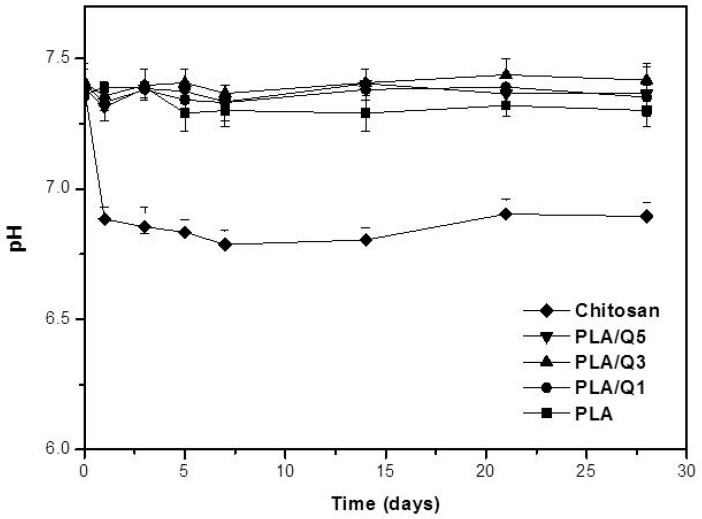
Changes in pH of PBS solution after different immersion times of PLA, chitosan, and PLA/chitosan composites with 1 wt % (PLA/Q1), 3 wt % (PLA/Q3), and 5 wt % (PLA/Q5) chitosan.

The effect of PLA, chitosan, and the composites in the pH of PBS as a function of the immersion time, is shown in Figure 5. It shows that the immersion of chitosan induces a decrease of the pH medium from ~7.4 to ~6.8 from the first day, and it varies relatively little thereafter. The change in pH of the PBS solution is negligible when the PLA and the composites are immersed, and it remains relatively constant at ~7.4 to 7.3.

The composites and the PLA are hydrolytically stable, since the immersion tests in PBS showed only a slight change in the pH values that remain in the range of 7.4–7.3. On the other hand, immersion of chitosan decreased the pH value to 6.9, because it has cationic properties [18,19,38] with low charge density, and ~60% of the NH_3_^+^ groups are deprotonated in neutral or basic medium; however there are electrostatic interactions between the anions present in the medium phosphate and the amino groups from the chitosan, which provoke the pH change of the solution medium. Similar behavior has been observed in molecules with phosphate groups and chitosan [38,39]. The current results show that the composites obtained by extrusion using commercial PLA and a chitosan extracted from shrimp waste are suitable for biological applications. These results coincide with other research that obtained PLA/chitosan composites by a different fabrication process [22,23].

#### 3.4.2. Adhesion of MG-63 Osteoblasts on the Composites PLA/Chitosan

**Figure 6:**
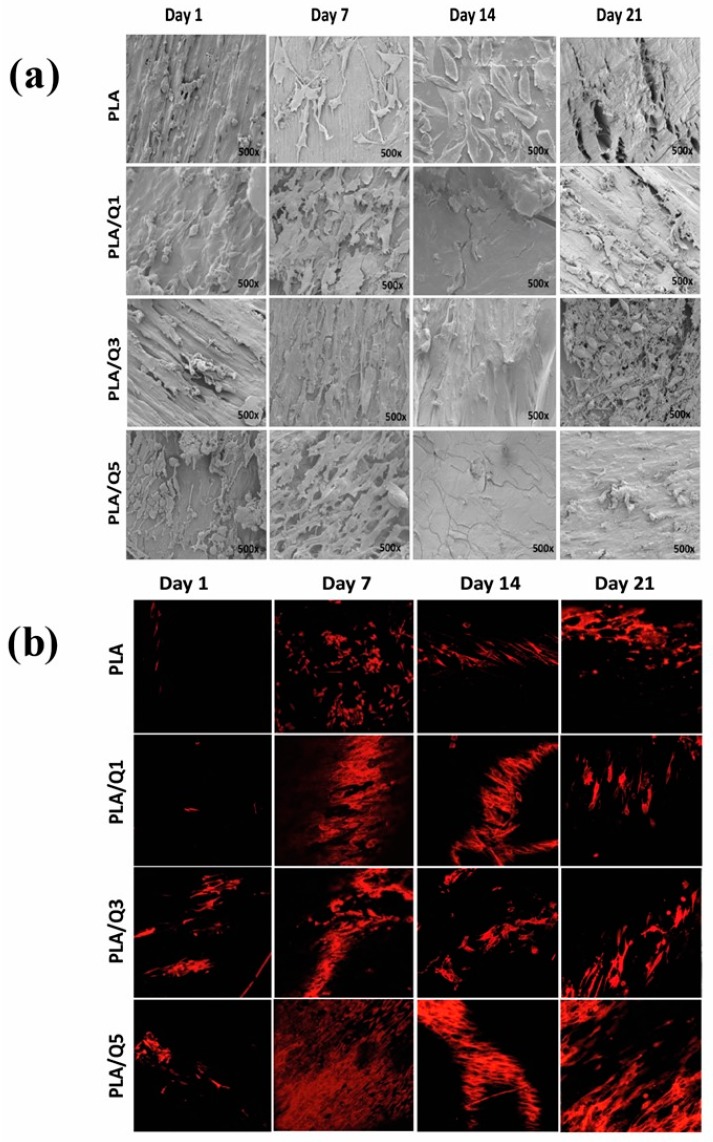
Adherence of MG-63 osteoblasts on PLA and PLA/chitosan composites after different incubation times. (**a**) SEM images taken at 500×; (**b**) Confocal microscopy images taken at 20×, the actin cytoskeleton is shown in red.

The adhesion and distribution of the MG-63 osteoblast cells on the PLA and the composites surface after different incubation times were studied by SEM and Confocal Microscopy; the images are visualized in Figure 6a,b, respectively. The PLA surface shows a good cell distribution and proliferative cell population that starts slowly from the first day, and increases with time. The cell adhesion and proliferation on the PLA/Q1, PLA/Q3, and PLA/Q5 composite surfaces advances faster than that of a single PLA, and it is quicker as the chitosan content increases. After 7 days, the surfaces of the PLA/Q3 and PLA/Q5 composites are almost completely covered, and this condition is maintained until the end of the kinetic tests. The cells adhered on PLA, and composites showed a normal cytoskeletal rearrangement with actin filaments longitudinally distributed. The SEM analysis shows that the adhered cells maintained the osteoblasts characteristic ultrastructural morphology. The cells were elongated with membranal prolongations that allowed them to join to the biomaterial surface.

The biological studies show that the MG-63 osteoblast cells are well adhered on the PLA and on the composites surfaces, and they present a characteristic and typical morphology that consists of membrane projections, some filopodia, and actin filaments longitudinally distributed. From the first test day, many cells adhered were viable and proliferate on the materials surfaces; this is due to the hydrophilic character of chitosan that influences cell adhesion and proliferation [40,41]. Among the three composites, the composites with 1 wt % (PLA/Q1) and 3 wt % (PLA/Q3) chitosan show greater osteoblast growth with respect to single PLA and to the composite with 5 wt % chitosan (PLA/Q5). Similar results have been reported with bone marrow mesenchymal stem cell (BMSC) on polylactic acid/chitosan obtained by grafting, in which the increase of the ratio of chitosan/lactic acid favors a higher number of BMSC cells [22]. Some authors have reported a low number of cells, and variation in their viability, density, and distribution [42]. These variations are due to different cell types and composites fabrication methods; this has been attributed to electrostatic interactions with negatively charged molecules such as glycosaminoglycans that permit to retain and concentrate the growth factors that are secreted by the osteogenic cells, exerting a chemotactic effect and increasing the osteoconduction [43,44].

#### 3.4.3. Viability and Proliferation of MG-63 Osteoblasts on the PLA/Chitosan Composites

**Figure 7:**
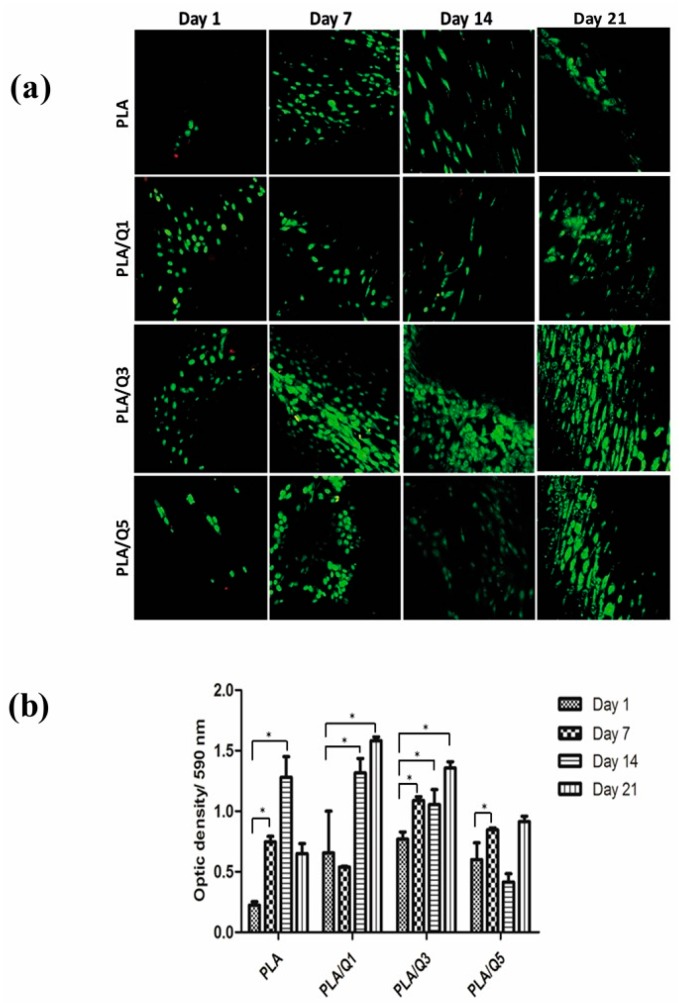
Viability and proliferation of osteoblasts on the PLA and PLA/chitosan composites surfaces. (**a**) Fluorescence of osteoblasts on PLA and composites. Viable cells are visualized in green and death cells are in red. Images were taken at 20×; (**b**) MTT metabolic activity of the osteoblasts on PLA and composites.

The viability of the cells adhered on the PLA and the biocomposites surface was analyzed qualitatively by fluorescence, and quantitatively by MTT assays. The results are presented in Figure 7a,b, respectively, where the viable cells are green and the non-viable ones are red. The PLA showed a low number of adhered cells compared with the composites, although the cells were viable. The three composites showed a high number of viable cells; only day 1 presented a small number of non-viable cells. These data are correlated with the quantitative results obtained in the MTT assays, and the results are shown in Figure 7b. Composites with 1 and 3 wt % chitosan show a maximum absorbance at 21 days, and they also present the highest values. For PLA and the composite with 5 wt % chitosan, the absorbance decreases after 14 and 7 days, respectively, because the available surface for cell proliferation is minimal due to the formed cell layers that already occupy the surface, as shown in Figure 6.

The biological tests therefore suggest the biocompatibility of PLA/chitosan composites, and they agree with the increase of the cell metabolic activity observed by MTT assays, promoting cell proliferation on the composite surface. In the case of the PLA/Q5 composite, metabolic activity decreases because the composite surface is completely covered with cells after only 7 days, and many cells die because of the limited surface that is still available for continuous growth, indicating that the cell death is not due to a cytotoxic effect, as has been described in other works [1,6,42,43].

#### 3.4.4. Qualitative Evaluation of Mineral Deposition on PLA/Chitosan Surfaces

**Figure 8:**
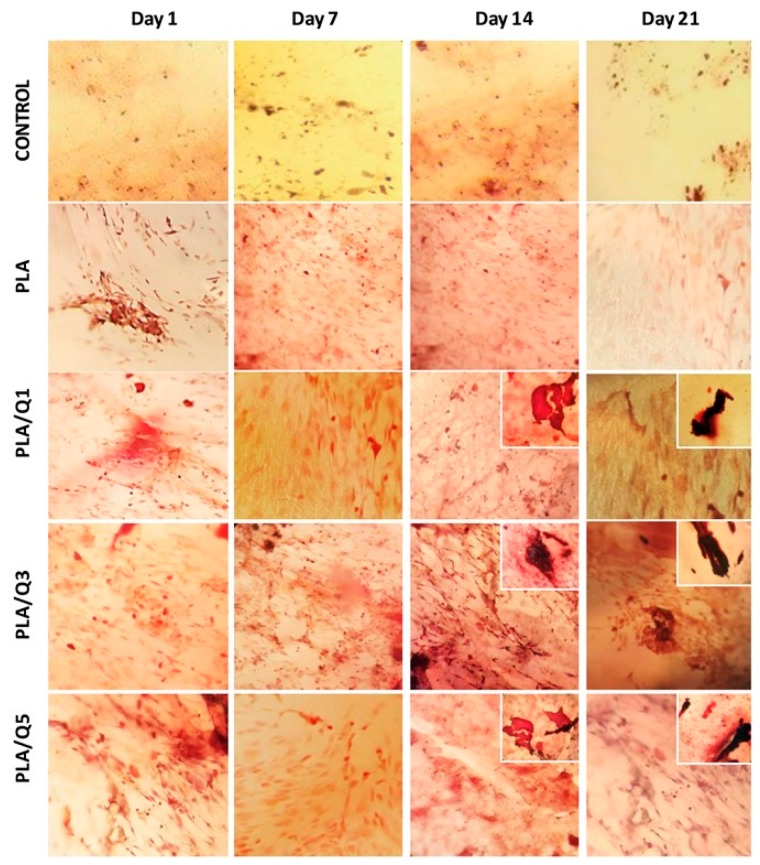
Osteoblasts grown on PLA and composites surfaces and mineralization. Cells were stained with Alizarin Red solution. Images were taken at 20×.

The presence of mineralized nodules on the surface of PLA and composites was evaluated qualitatively by staining the cells with an Alizarin red solution; the results are shown in Figure 8. The behavior that is observed for each material follows the same trend perceived in the other assays shown in Figure 6 and Figure 7. The PLA showed minimal mineralization at all evaluated test times. The PLA/chitosan composites showed a greater amount of mineral deposition, and presented an intense red staining, with nodules significantly bigger than those of PLA. This effect increases with time, starting on day 14, and is maintained until day 21. The chitosan addition to the composites significantly increases the mineralization. On day 14, there are many more mineralization nodules on the composite surfaces compared to PLA. The chitosan enhances the osteoblast mineralization due to its bioactive properties. The cells differentiate on the anionic matrix, absorbing calcium and phosphate ions, and then the calcification occurs by a nucleation and growth process [44,45].

These results suggest that the PLA/chitosan composites can support the growth of bone tissue. However, it is necessary to investigate the expression of several proteins, such as alkaline phosphatase, osteopontin, osteonectin, and osteocalcin, among others, and some transcription factors like Runx2, to establish the osteoinductive characteristic of the composites.

## 4. Conclusions

Composites of polylactic acid with 1, 3, and 5 wt % chitosan have been fabricated without the use of solvents by extrusion of chitosan particles extracted from shrimp exoskeletons and PLA, for the purpose of improving the biocompatibility of PLA alone. The investigation shows that the raw materials, composition, and fabrication with a low cost process implemented is reproducible, and does not compromise the biocompatibility of the biocomposites. The composites are non-toxic, and increasing the chitosan content favors cell adhesion, proliferation, and metabolic activity on the composites’ surface. The composites are appropriate for the growth of MG-63 osteoblast cells, and further cell reproduction and mineralization. Their mechanical properties are suitable for biomaterial applications. The PLA/chitosan composites with 1–5 wt % chitosan are potential candidates for applications in tissue engineering, for use as scaffolds.

## Figures and Tables

**Table 1:** Relevant mechanical properties of Polylactic acid (PLA) and Polylactic acid/chitosan composites with different amounts of chitosan.

Sample	Amount of Chitosan (wt %)	Tensile Strenght (MPa)	Young’s Modulus (MPa)	Elongation at Break (%)
PLA	–	49.36 ± 4.44	1252 ± 62	4.97 ± 0.76
PLA/Q 1	1	56.34 ± 2.64	1110 ± 66	7.12 ± 0.63
PLA/Q 3	3	43.38 ± 3.29	983 ± 55	6.63 ± 0.54
PLA/Q 5	5	42.19 ± 2.55	1051 ± 34	6.52 ± 0.86
